# Rise and Decay of Photoluminescence in Upconverting Lanthanide-Doped Nanocrystals

**DOI:** 10.1021/acsnano.4c09945

**Published:** 2024-10-05

**Authors:** Sander J. W. Vonk, J. J. Erik Maris, Ayla J. H. Dekker, Jur W. de Wit, Thomas P. van Swieten, Ario Cocina, Freddy T. Rabouw

**Affiliations:** † Soft Condensed Matter & Biophysics, Debye Institute for Nanomaterials Science, Utrecht University, Princetonplein 1, 3584 CC Utrecht, The Netherlands; ‡ Inorganic Chemistry & Catalysis, Debye Institute for Nanomaterials Science & Institute for Sustainable and Circular Chemistry, Utrecht University, Universiteitsweg 99, 3584 CG Utrecht, The Netherlands; § Organic Chemistry & Catalysis, Institute for Sustainable and Circular Chemistry, Utrecht University, Universiteitsweg 99, 3584 CG Utrecht, The Netherlands; ∥ Optical Materials Engineering Laboratory, ETH Zürich, Leonhardstrasse 21, 8092 Zürich, Switzerland

**Keywords:** colloidal nanocrystals, upconversion, lanthanide ions, excited-state dynamics, local density of optical states

## Abstract

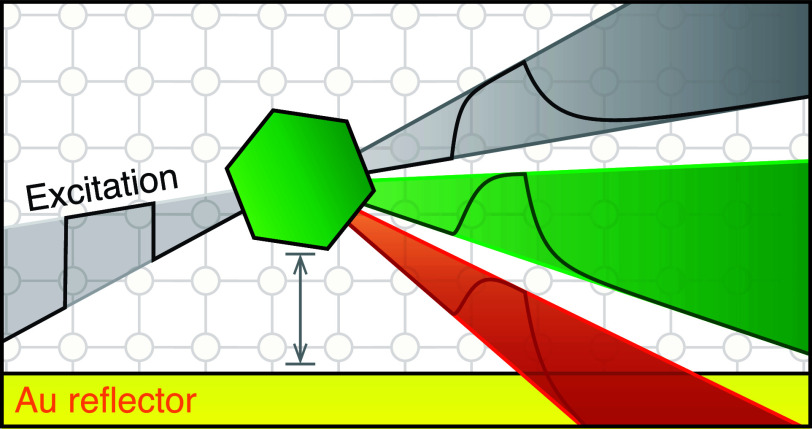

Nanocrystals (NCs) doped with lanthanides are capable of efficient photon upconversion, i.e., absorbing long-wavelength light and emitting shorter-wavelength light. The internal processes that enable upconversion are a complex network of electronic transitions within and energy transfer between dopant centers. In this work, we study the rise and decay dynamics of upconversion emission from β-NaYF_4_ NCs codoped with Er^3+^ and Yb^3+^. The rise dynamics of the red and green upconverted emissions are nonlinear, reflecting the nonlinear nature of upconversion and revealing the mechanisms that populate the emitting states. The excited-state decay dynamics are nonexponential. We unravel the underlying decay pathways using photonic experiments. These reveal the contributions of different upconversion pathways visually, as each pathway exhibits a distinct response to systematic variation of the local density of optical states. Moreover, the effect of the local density of optical states on core-only NCs is qualitatively different from core–shell NCs. This is due to the different balance between feeding and decay of the electronic levels that produce upconverted emission. The understanding of the upconversion dynamics provided here could lead to better imaging and sensing methods relying on upconversion lifetimes or guide the rational optimization of the dopant concentrations for brighter upconversion.

## Introduction

Upconverting nanocrystals (NC) are a promising class of color converters that transform low-energy into higher-energy light. Owing to their rich energy-level structure,^1^ lanthanide ions doped into NCs are ideal for upconversion, as their electronic transitions facilitate both absorption of low-energy and emission of high-energy photons. Common material designs use Yb^3+^ sensitizer ions (strongly absorbing), which transfer their energy to Er^3+^ or Tm^3+^ activator ions (supporting high-energy electronic transitions). These ions are typically codoped in inorganic NaYF_4_, which is a popular host material with minimal nonradiative losses because of its low phonon energy.^2^ Colloidal upconverting NCs offer the advantage of solution processability, which facilitates incorporation into many applications such as anticounterfeiting ink,^3^ background-free imaging in biological systems,^4^ solar cells to maximize light-to-energy conversion,^5^ and background-free optical sensing of temperature,^6^ pressure,^7^ and the chemical environment.^8^

The light output of upconversion NCs is determined by complex excited-state dynamics that depend on various materials properties and external parameters. Reports in the past have studied the excited-state pathways experimentally, and successfully explained and/or reproduced trends observed using theoretical models.^9−21^ Most commonly, rate-equation models are used that include processes such as photoabsorption and -emission, multiphonon relaxation (MPR), cross relaxation (CR), energy transfer (ET), migration, and energy-transfer upconversion (ETU). The steady-state solutions to these models are typically compared to experimental trends such as the power dependence of upconversion emission intensity.^21−23^ While such comparisons provide useful insights, they do not pose a strict test for the assumptions on the excited-state pathways nor for the many input parameters of the model. Fundamental understanding and predictive modeling would benefit from more focused experiments that zoom in on individual excited-state pathways and their contribution to upconversion emission. This in turn could aid the development of upconverting schemes and materials.

In this Article, we investigate the rise and decay dynamics of upconverted emission from Er^3+^ and Yb^3+^ codoped NaYF_4_ NCs. While previous systematic experiments used low-power and resonant excitation into the emitting levels of such particles,^24^ here we study the excited-state pathways upon high-power upconversion excitation. The rise dynamics of the direct near-infrared (NIR, 1000-nm emission) emission and the green and red upconversion emissions are strongly power-dependent and reveal NIR photoabsorption by Yb^3+^ as the rate-limiting step. The upconversion decay dynamics are power-dependent and multiexponential. We obtain insights into which levels are involved in upconversion from photonic experiments. To distinguish and assign different contributions we systematically vary the photonic environment,^25^ which modulates the rates of all radiative transitions while nonradiative transitions are unaffected.^26^ Surprisingly, we find a significant signature of the ^4^I_13/2_ → ^4^I_15/2_ transition rate in the green and red upconversion decay of core–shell NCs, which reveals the importance of higher-order ETU involving the ^4^I_13/2_ level. Comparing the dynamics of core-only and core–shell NCs, we observe a crossover from decay-limited (core-only NCs) to feeding-limited (core–shell NCs) upconversion decay dynamics. Our results highlight the importance of in-depth characterization of all decay pathways at high excitation powers to gain a fundamental understanding of upconverting materials and their excited-state dynamics.

## Results and Discussion

### Comparing Upconverting Materials to Two-Level Systems

**Figure 1:**
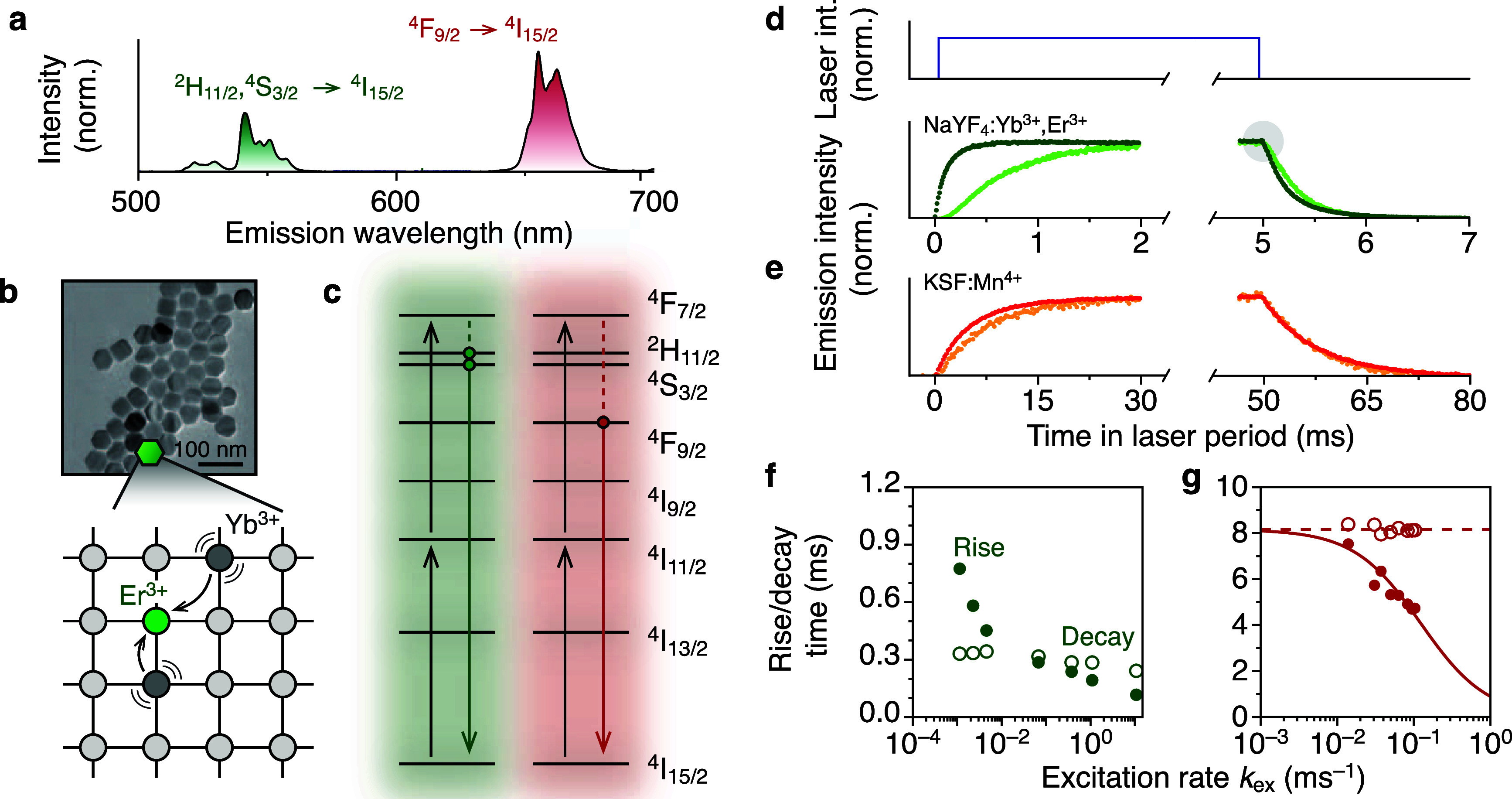
Upconverting nanocrystals are not two-level systems. (a) Upconversion emission spectrum of β-NaYF_4_ core-only NCs doped with Yb^3+^ and Er^3+^with bright green and red emission. (b) Electron-microscopy image of core-only hexagonal β-NaYF_4_ NCs. In the NaYF_4_ host, a fraction of the Y^3+^ ions is replaced by Yb^3+^ and Er^3+^. After 980-nm absorption, Yb^3+^ can transfer their excess energy to a nearby Er^3+^ ion. (c) Simplest scheme for red and green upconversion for Er^3+^, where 2 energy-transfer steps (black upward arrows) drive Er^3+^ to the ^4^F_7/2_ energy level. Multiphonon relaxation populates the green- and red-emitting levels. (d) Rise and decay of core-only NaYF_4_:Yb^3+^,Er^3+^ for 980 nm 5 ms block pulses at low (light green, *k*_ex_ = 10^–3^ ms^–1^) and high (darker green, *k*_ex_ = 10 ms^–1^) excitation rates. Both rise and decay are faster for higher excitation rates. We observe “postponed decay” at low power (inset, light green), i.e. the emission intensity remains relatively stable after the laser is turned off before it drops, indicating continued feeding after the laser is off. (e) Same as (d), but for K_2_SiF_6_:Mn^4+^ which behaves as a two-level system at low (orange, *k*_ex_ = 10^–2^ ms^–1^) and high (red, *k*_ex_ = 10^–1^ ms^–1^) power (Reproduced from data presented in ref (29)). Here, contrasting with the upconversion signatures in (d), the decay dynamics are independent of power. (f) Rise (1–e^–1^ times) and decay time (e^–1^ times) of the green upconversion emission from core-only β-NaYF_4_:Yb^3+^,Er^3^ as a function of excitation rate *k*_ex_. (g) Same as (f), but for K_2_SiF_6_:Mn^4+^ showing characteristic power-dependent rise and decay times for 2-level system. Dashed red line: average decay rate *k*_2_ over all excitation powers. Solid red line: rise time τ_rise_ = (*k*_ex_ + *k*_2_)^−1^ with the average decay rate as input. Data reused from ref (29).

We study upconverting β-NaYF_4_ NCs codoped with Yb^3+^(18%) and Er^3+^(2%), which show bright green and red upconversion emission (Figure 1a, core-only sample) upon 980-nm excitation (Supporting Information section S1 for TEM images of all samples used in this work). In this inorganic nanohost, Y^3+^ ions are randomly replaced by Yb^3+^ and Er^3+^ ions (Figure 1b). Sensitizer Yb^3+^ ions can absorb 980-nm light and subsequently transfer their energy to a nearby Er^3+^ ion. In the simplest picture of upconversion, consecutive energy transfer (ET) and energy-transfer upconversion (ETU) drive Er^3+^ to the ^4^F_7/2_ energy level, which after MPR precedes green (^2^H_11/2_,^4^S_3/2_ → ^4^I_15/2_) and red (^4^F_9/2_ → ^4^I_15/2_) emissions. Figure 1c shows the simplest two-photon upconversion pathways, but more complex pathways are also operative.

Figure 1d shows the rise and decay of green upconversion emission for core-only NCs upon excitation with 980-nm 5-ms block pulses at different excitation powers. Both rise and decay accelerate at higher powers. Figure 1f shows that the upconversion rise is slower than decay at low power, but faster at higher power [quantified in terms of the 1–e^–1^ (rise) and e^–1^ (decay) times]. This behavior is markedly different from reference experiments of the rise and decay dynamics of a Mn^4+^-based phosphor (Figure 1e). Here, the observations are the textbook example of a 2-level system (Supporting Information section S2): the decay time—after the laser is turned off—is independent of laser power, while the rise time becomes faster with increasing laser power as τ_rise_ = (*k* + *k*_ex_)^−1^, where *k* is the decay rate and *k*_ex_ is the excitation rate (Figure 1g).

The upconversion emission dynamics (Figure 1d,f) are different than those of a 2-level system (Figure 1e,g). The initial rise of upconversion emission at low power is superlinear (*t* < 0.5 ms in Figure 1d, light green) indicating that feeding of the upconversion emission is a multistep process, e.g. absorption, ET, and ETU. Moreover, we observe “postponed decay” at low power, i.e. the emission intensity does not drop immediately after the laser is turned off but instead remains high during the first 0.1 ms (Figure 1d, inset shows zoom-in of *t* = 4.8–5.2 ms). This indicates continued feeding of the green-emitting level while the excitation laser is off.

To understand the complex upconversion dynamics in Figure 1, a common strategy would be to study samples with different doping concentrations. Although this can yield useful insights, quantitative interpretations are difficult because many rate constants change simultaneously with doping concentration, including those for CR, ETU, and energy migration. Moreover, different batches of NCs have inevitable differences in particle size and defect concentration. Therefore, we use a different procedure. In what follows, we will systematically vary the photonic environment while keeping the same sample. In this way, we keep CR, MPR, and energy-migration rates constant,^27,28^ but tune the rates of absorption and spontaneous emission, allowing us to distinguish different decay processes.^25^

### NIR-Emitting Feeding Levels for Upconversion

**Figure 2:**
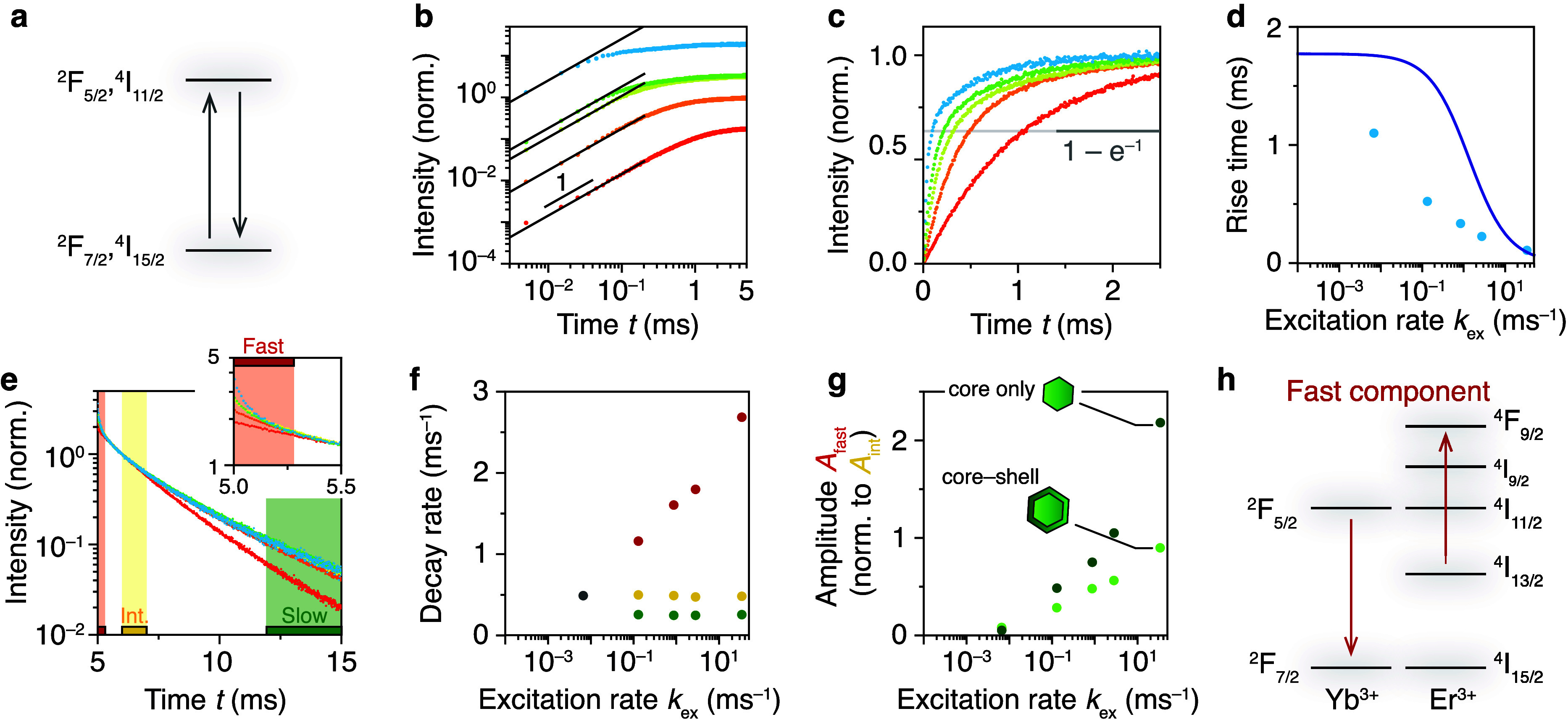
Rise and decay of the NIR-emitting feeding levels in core–shell NCs. (a) By exciting at 980 nm and detecting in the NIR, we probe the ^2^F_5/2_ level of Yb^3+^ and ^4^I_11/2_ level of Er^3+^ simultaneously. (b) Rise curves a log–log scale as a function of excitation power (increasing from red to blue) show a linear initial rise in time for all powers, consistent with the expected dynamics for a two-level system. (c) Same as (b) but normalized and with a linear *y*-scale. The rise time is defined as the time where the normalized rise curves cross 1–e^–1^. (d) Extracted rise times from panel (c) (blue dots) are unexpectedly short compared to the expectations for a two-level system (solid line), indicating additional depopulation channels from the NIR-emitting levels on top of radiative decay. (e) Excited-state decay of the NIR-emitting levels after the laser is turned off (normalized to *t* = 6 ms, excitation rates same as (b,c) increasing from red to blue). At the lowest excitation rate (*k*_ex_ = 4 × 10^–3^ ms^–1^), we observe single-exponential decay with a decay rate *k* = 0.49 ms^–1^. At higher excitation rates, 2 additional fast (zoom-in on first 0.5 ms as an inset), and slow decay-curve components appear. (f) Decay rate of the 3 selected decay-curve components in (e) as a function of excitation rate [bars in panel (e) show the fit ranges]. The decay rate of the fast component (red dots) increases with excitation rate and the intermediate (yellow) and slow (green) decay are approximately independent of excitation power. Gray dot: decay rate for the single-exponential decay at the lowest excitation rate. (g) The relative amplitude of the fast decay component as a function of excitation rate for core-only (darker green) and core–shell (lighter green) NCs. Fast decay is more pronounced in core-only NCs. (h) Proposed excited-state decay responsible for the fast-decaying component in (e). ETU from the ^2^F_5/2_ to the ^4^I_13/2_ level (red arrows) feeds the red-emitting ^4^F_9/2_ energy level of Er^3+^. This ETU process is expected to be faster in core-only NCs because of the higher population of Er^3+^ ions in the ^4^I_13/2_ level due to fast MPR assisted by vibrations on the NCs’ surface.^31^

We start by studying the rise and decay dynamics of the NIR-emitting levels in core–shell NaYF_4_:Yb^3+^,Er^3+^ NCs (Figure 2a), which are the feeding levels for both red and green upconverted emission. By exciting at 980 nm and detecting in the NIR using a 1000-nm long-pass filter, we probe the ^2^F_5/2_ level of Yb^3+^ and ^4^I_11/2_ level of Er^3+^ simultaneously. We cannot distinguish between these levels, because they emit at the same energy and rapidly exchange energy.^30^ Most of the emission will originate from the ^2^F_5/2_ level, because it has the faster radiative decay rate and the doping concentration of Yb^3+^ is 9× higher than Er^3+^. Figure 2b shows the rise dynamics of the NIR emission for over 4 orders of magnitude in excitation rate (*k*_ex_ = 10^–3^–10^1^ ms^–1^, determined from the measured illumination intensity and the known absorption cross section of Yb^3+^ in NaYF_4_; see Method section for details). The initial rise over the first <0.1 ms is linear for all excitation powers, showing that the emitting level is populated by a simple one-step photon-absorption process (Supporting Information section S2). On a longer time scale, we observe that the rise time τ_rise_—defined as the time at which the normalized intensity crosses 1–e^–1^—decreases with excitation rate (Figure 2c; same data as Figure 2b but normalized). This behavior is qualitatively similar to the two-level system presented in Figure 1e. However, the rise times do not follow the power dependence of τ_rise_ = (*k*_ex_ + *k*_NIR_)^−1^ expected for a 2-level system (Figure 2d), using the radiative decay rate *k*_NIR_ = 1/(1.72 ms) of Yb^3+^.^24^ The rise times are unexpectedly short, which highlights the presence of extra depopulation channels—for example ETU—in addition to radiative decay.

We follow the excited-state decay of the NIR levels after the laser is turned off (Figure 2e). To obtain a high dynamic range in our measurements (over detector dark counts), we maximize the signal rate hitting our single-photon detectors and correct for missed photon-detection events due to the detector deadtime (Supporting Information section S3). We exclude effects of laser heating in our experiment by recording the Er^3+^ emission spectrum in the green, which is temperature-sensitive but reveals negligible heating (Figure S4). At a low excitation rate (Figure 2e, red; *k*_ex_ = 4 × 10^–3^ ms^–1^), we observe single-exponential decay. At higher excitation rates, faster (red bar) and slower (green bar) decay components appear in the decay curve (Figure 2e). The decay rate of the fast-decaying component increases with increasing excitation power, while the characteristic times of the other components are approximately constant (Figure 2f, single-exponential fit to time ranges indicated in Figure 2e). We attribute the increasingly dominant fast component to ETU, as the rate of ETU increases with excitation rate because of growing excited-state populations. Comparing the NIR decay dynamics of core–shell (Figure 2e,f) to core-only (Supporting Information Figure S5) NCs reveals that the fast component has a larger relative amplitude in core-only NCs (Figure 2g, dark green). Based on this trend, we hypothesize that ETU from the ^2^F_5/2_ level of Yb^3+^ to excite Er^3+^ from ^4^I_13/2_ to ^4^F_9/2_—not the conventional ETU that excites Er^3+^ to the ^4^F_7/2_ level—is an important contributor to the fast component of the NIR decay (Figure 2h). This pathway explains the more pronounced ETU feature in the excited-state dynamics of core-only NCs, as boosted ^4^I_11/2_ → ^4^I_13/2_ MPR by surface-related vibrations leads to higher population of the ^4^I_13/2_ level compared to core–shell NCs.^31^

**Figure 3:**
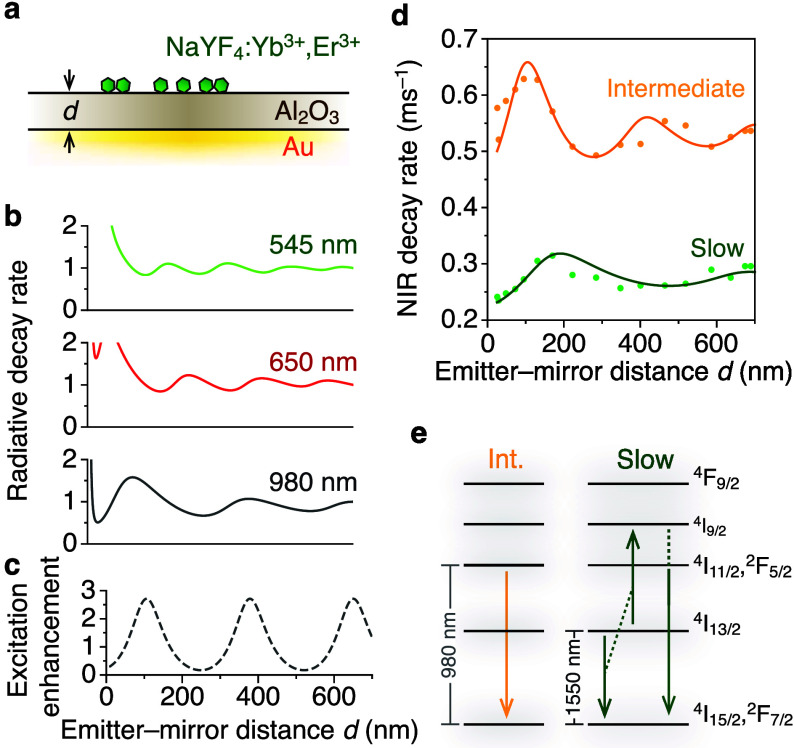
Investigating the origin of different NIR decay components using a photonic approach. (a) Ramped-reflector substrate consisting of a Al_2_O_3_ spacer layer with spatially varying thickness *d* separating the upconverting core–shell NCs from a Au reflector. (b) The calculated radiative decay rates for the green-, red-, and NIR-emitting levels vary over the ramped-reflector substrate because of varying local density of optical states (LDOS). The branching ratios of longer-wavelength emissions to excited states and magnetic-dipole contributions are taken into account. (c) The local 980-nm excitation enhancement (at constant power setting, relative to infinite emitter–mirror distance) varies over the substrate because of constructive or destructive interference of the laser light near the Au reflector. The oscillations of excitation enhancement are different from the those of radiative decay at 980 nm (panel b) because the excitation light comes in from a finite range of angles, while emission goes in all directions. (d) Excited-state decay rate of the intermediate (orange) and slow (green) decay component of NIR emission on the ramped-reflector substrate after 2-ms-pulsed 980-nm excitation. The excitation rate is approximately *k*_ex_ = 0.1 ms^–1^ at infinite distance from the Au reflector (Method section for details). For the intermediate component, the oscillations match the oscillations expected for direct decay from the Er^3+^^4^I_11/2_ and Yb^3+^^2^F_5/2_ levels. We model the intermediate decay rate as *k*_NIR_^int^(*d*) = *k*_nr,NIR_^int^ + *k*_r,NIR_^int^ρ(980 nm, *d*), using the LDOS at 980 nm, and by fitting the nonradiative decay rate *k*_nr,NIR_^int^ = 0.33 ms^–1^ and radiative decay rate *k*_r,NIR_^int^ = 0.12 ms^–1^. For the slow component *k*_NIR_^slow^, the oscillations have a longer periodicity than any of the expected green, red, NIR LDOS for upconverting materials, indicating that a radiative transition with a smaller energy gap is involved in the excited-state decay. We find a good fit to *k*_NIR_^slow^(*d*) = *k*_nr,NIR_^slow^ + *k*_r,NIR_^slow^ρ(1550 nm, *d*), using the 1550 nm LDOS, fitting a nonradiative decay rate *k*_nr,NIR_^slow^ = 0.15 ms^–1^ and radiative decay rate *k*_r,NIR_^slow^ = 1.6*k*_r, IR_^0^, where *k*_r, IR_^0^ = 0.04 ms^–1^ is the radiative decay rate of the IR emitting level of Er^3+^. (e) Schematic excited-state decay leading to the intermediate (left) and slow (right) decay components of NIR emission. The intermediate decay component is regular direct decay of ions in the NIR-emitting levels. For the slow decay component, the LDOS dependence revealed feeding by ETU from the ^4^I_13/2_ level.

Next, we investigate the intermediate NIR decay component, which is present at low power and remains at increasing powers (yellow in Figure 2e) and the slow NIR decay component, which becomes apparent at higher powers (green in Figure 2e). To reveal the origin of the these decay components, we use a photonic approach by placing a monolayer of upconverting core–shell NCs on a ramped-reflector substrate consisting of a Au reflector and a ramped Al_2_O_3_ spacer.^25^Figure 3a shows a schematic of the geometry and Figure S5 shows a scanning electron microscopy image of a monolayer of NCs on a spacer. The radiative decay rates of Er^3+^ and Yb^3+^ are modulated by interference that affects the local density of optical states (LDOS) ρ. As different levels emit different wavelengths of light, the interference effects on the levels depend differently on the distance *d* between the emitters and Au. More precisely, the radiative decay rate of levels emitting longer-wavelength light oscillates with a longer periodicity than levels that emit shorter-wavelength light (Figure 3b). In addition, interference of our 980-nm laser creates a standing-wave pattern and modulates the excitation power over the ramped-reflector substrate with yet another distinct periodicity (Figure 3c, Supporting Information section S4). The different dependencies of radiative decay rates and excitation power on distance to the Au reflector will allow us to distinguish the excited-state pathways contributing to upconversion emission. Our calculations of Figure 3b,c, and our further analysis of LDOS dependencies presented below, account for the finite branching ratios of radiative transitions to excited states as well as magnetic-dipole contributions to the transitions (Supporting Information section S4).^32,33^ These wavelength-specific effects make photonic experiments on a reflector substrate different from photonic experiments using solvents of different refractive index, where radiative transition rates are changed by the same factor irrespective of the emission wavelength.^34^

Experimentally, we observe that the decay rates of the intermediate and slow NIR decay components (Figure 3d), after pulsed 980 nm excitation (2 ms pulse duration), vary over the ramped-reflector substrate, but with different periodicities. We exclude that the decay rates are modulated by excitation-power as the oscillations are clearly different from the one presented in Figure 3c and the power-dependent experiments (Figure 2e,f) showed no effect. The different periodicities of intermediate and slow decay indicate that different excited-state decay pathways precede NIR photon emission. Although we are certain that we detect only emission at NIR wavelengths—by using a 1000 nm long-pass filter and silicon detectors—the *lifetime-determining* step must involve levels that emit at different wavelengths.

We reproduce the decay rate *k* of the intermediate component vs *d* using a self-interference model. In this work, we label the extracted decay rates for the electronic transitions leading to green, red, near-infrared emission *k*_G_, *k*_R_, and *k*_NIR_, respectively. We fit the intermediate decay component *vs d* to *k*_NIR_^int^(*d*) = *k*_nr,NIR_^int^ + *k*_r,NIR_^int^ρ(980 nm, *d*), where ρ(980 nm, *d*) is the LDOS for 980-nm NIR emission (Figure 3b) and *k*_nr,NIR_^int^ and *k*_r,NIR_^int^ are the only fit parameters (Supporting Information Section S4 for all fitted decay rates in this work). The fit result is shown in Figure 3d. In this fitting routine, and in the rest of this Article, we always determine which of the possible LDOS functions [ρ(λ, *d*) for the different emitting levels with λ = {545,650,980,1550} nm or the excitation enhancement ρ_ex_(980 nm, *d*)] provides the best match with the smallest minimized error function (Figure S6). As discussed before, a good match between the 980-nm LDOS and our data shows that the intermediate decay component is due to regular direct decay of ions in the ^4^I_11/2_ (Er^3+^) and ^2^F_5/2_ (Yb^3+^) energy levels to the ground state (Figure 3e). The noise on the data may be due to variations in the Al_*x*_O_*y*_ stoichiometry, variations in roughness of the interfaces, uncertainties in local spacer thickness, or air voids in the spacer layer.

Interestingly, the oscillations in the slow decay component *k*_NIR_^slow^(*d*) have a longer periodicity (Figure 3d, green dots), indicating that radiative decay from an electronic transition with *lower* energy Δ*E* (longer emission wavelength) than the NIR levels is involved in the excited-state decay. Indeed, we obtain a good fit with the data using the LDOS at 1550 nm ρ(1550 nm, *d*), which is the emission wavelength of the lower-lying ^4^I_13/2_ → ^4^I_15/2_ transition, which we will call the “IR” emission in this Article. The photonic experiments thus show that the slow NIR emission dynamics arise because of slow feeding from the ^4^I_13/2_ level by ETU, i.e. ^4^I_13/2_ + ^4^I_13/2_ → ^4^I_9/2_ → ^4^I_11/2_ (Figure 3e). To study this excited-state pathway in more depth, we approximate the differential equation for the NIR population *n*_NIR_^slow^ that is due to ETU from the IR level by
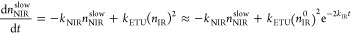
1where *k*_ETU_ is the rate constant for ETU, *n*_IR_ is the population of the IR level, superscript 0 indicates the value at *t* = 0 when the block pulse is turned off, and *k*_NIR_ and *k*_IR_ are the total decay rates from the NIR- and IR-emitting levels, respectively. If we assume steady-state populations after the block pulse, such that *n*_NIR_^slow,0^ = *k*_ETU_(*n*_IR_^0^)^2^/*k*_NIR_, the decay is as follows:
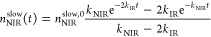
2

For our NCs, *k*_IR_ ≪ *k*_NIR_, and we observe the slow component at *k*_NIR_*t* > 1. In this regime, *n*_NIR_^slow^(*t*) decays approximately exponentially with a rate of 2*k*_IR_, where the factor of 2 originates from the quadratic dependence of the feeding on IR population. Indeed, the decay rate of the slow NIR component matches well with the model *k*_NIR_^slow^(*d*) = *k*_nr, NIR_^*s*low^ + *k*_r, NIR_^*s*low^ρ(1550 nm, *d*), with *k*_r, NIR_^*s*low^ = 1.6*k*_r, IR_^0^ (Figure 3d, green line). Here, *k*_r, IR_^0^ is the radiative decay rate of the ^4^I_13/2_ level at infinite distance from the Au reflector (Supporting Information Section S4). The surprising LDOS oscillations in the slow NIR component are hence fully consistent with ETU feeding from the ^4^I_13/2_ level. The extracted radiative decay rate of the intermediate decay component *k*_r,NIR_^int^ is however somewhat smaller than expected, i.e *k*_r,NIR_^int^ = 0.61*k*_r,NIR_^0^. This might indicate the difficulty of isolating direct decay from the slow decay involving ETU processes, damping the oscillations.

### Rise and Decay of Upconverted Emission

**Figure 4:**
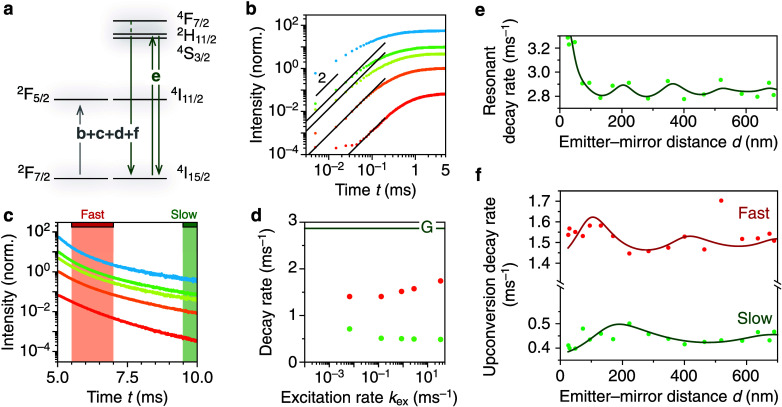
Rise and decay of green upconverted emission in core–shell NCs. (a) Upconversion excitation leads to green emission after ET and ETU (panels b–d,f). Alternatively, we can directly extract the excited-state dynamics of the green-emitting level using resonant 520-nm excitation (panel e). (b) Rise curves of green upconverted emission on a log–log scale for 980-nm excitation (pulse duration of 5 ms) for over 4 orders of magnitude in excitation rate (*k*_ex_ = 10^–2^–10 ms^–1^, from red to blue). We observe an approximately quadratic rise at the lowest 4 excitation rates, indicating two slow photon-absorption steps in the upconversion feeding process (Supporting Information section S5). At the highest excitation rate (blue), we observe a subquadratic rise, indicating that even photon absorption is not rate-limiting on the time scales of our experiment. (c) Decay curves of upconverted green emission after the laser is turned off. We observe biexponential decay with fast (red range) and slowly decaying components (green range). (d) Extracted decay rates by fitting single-exponential decay to the indicated ranges in (c) for the fast (red) and slow (green) components. The decay rates are approximately constant with excitation rate and are slower than direct decay from the green level after resonant excitation (green line). (e) The resonant decay rate of green emission after 520-nm excitation is modulated by the total LDOS at 545 nm on the ramped-reflector substrate. We fit the data to our model *k*_G_(*d*) = *k*_nr,G_ + *k*_r,G_^0^ρ(545 nm, *d*), and fit a radiative decay rate *k*_r,G_^0^ = 0.70 ms^–1^ and nonradiative decay rate *k*_nr,G_ = 2.02 ms^–1^. The nonradiative decay rate is high because of CR. (f) Upconversion decay rates of the fast (red dots) and slow (green dots) decay components on the ramped-reflector substrate after 2-ms-pulsed 980-nm excitation. The components follow the total LDOS at 980 nm (fast) and 1550 nm (slow), just as the intermediate and slow components of NIR emission (Figure 3d). The ETU rate is proportional to [*n*_NIR_(*t*)]^2^. As both 2*k*_NIR_ and 4*k*_IR_ are much slower than *k*_G_, we observe the decay rates of the NIR feeding level in the upconversion-emission decay. For the fast component we fit *k*_G_^fast^(*d*) = *k*_nr,G_^fast^ + 0.57*k*_r,NIR_^0^ρ(980 nm, *d*). The prefactor in front of *k*_r,NIR_^0^ is smaller than the value of 2 presumably because of the overlap with direct green emission and the slower decay component in the fitted time range. For the slow component, we successfully fit *k*_G_^slow^(*d*) = *k*_nr,G_^slow^ + 2.1*k*_r,IR_^0^ρ(1550 nm, *d*) to the data, with a prefactor in front of *k*_r, IR_^0^ close as to what is expected for a four-photon ETU process.

With a better understanding of the excited-state dynamics of the NIR feeding levels, we investigate the rise and decay of green upconverted emission (Figure 4a). Figure 4b shows the rise of green emission for 980-nm excitation on a log–log scale over 4 orders of magnitude in excitation rate (same excitation rates as in Figure 2). We observe that the initial rise is quadratic, i.e. intensity *I* ∝ *t*^2^, for the lowest 4 excitation rates (Figure 4b, solid lines). This is perhaps surprising, as the exponent of the initial rise should reflect the number of electronic processes that lead to population of the emitting level (Supporting Information section S5). One would expect that green upconversion emission is preceded by as many as five steps: two photon-absorption processes by Yb^3+^, ET from Yb^3+^ to Er^3+^, ETU to the ^4^F_7/2_ level, and MPR. Apparently, only two of these processes are slower than the 5-μs time resolution of our experiment, while faster processes than the time resolution do not contribute to the rise exponent (Supporting Information section S5). These two slow processes must be the photon absorption events by Yb^3+^, because the excitation-rate constants are slower than the time resolution of our experiment. At the highest excitation rate we observe a subquadratic rise, indicating that even photon absorption becomes less rate-limiting for the rise of green upconversion emission. It is important to note here that the early rise dynamics (<100 μs) of the green upconversion emission are likely dominated by the subset of Er^3+^ and Yb^3+^ ions that are closest to each other in the NaYF_4_ lattice and most strongly coupled through ET and ETU. The rate constants for ET and ETU between these ions may be faster than 5 μs but the average values could be slower. We observe qualitatively similar results for green upconverted emission in core-only NCs (Supporting Information Figure S8). For red upconversion emission, we observe a cubic rise (*I* ∝ *t*^3^) at the lowest excitation rates, indicating an additional slow photon-absorption process involved [Supporting Information Figure S8 (core-only NCs) and Figure S9 (core–shell NCs)]. This is consistent with a previously reported possible three-photon feeding pathway, where CR from the green level populates the ^4^I_13/2_ level from which ETU results in population of the red-emitting level.^9^

Figure 4c shows the excited-state decay of the green-emitting levels of Er^3+^. We observe biexponential decay consisting of fast (red bar) and slow (green bar) decay components with decay rates approximately independent of the excitation rate (Figure 4d). Interestingly, both decay components are long-lived compared to green emission after resonant excitation (Figure 4d, gray line), but short-lived compared to the NIR decay components from Figures 2 and 3. The decay rate of green emission after resonant excitation on the ramped-reflector substrate follows the LDOS at the emission wavelength around 545 nm (Figure 4e). By fitting *k*_nr,G_ = 2.02 ms^–1^, we extract an emission efficiency η_G_ = 26%, which is low because of CR and consistent with previous studies on NCs with 18% Yb^3+^ and 2% Er^3+^.^24^ Interestingly, *upconverted* green emission (Figure 4f) has a different periodicity compared to resonant excitation (Figure 4e). In fact, the oscillations observed for the green upconversion decay rates (Figure 4f) are similar to those for the intermediate and slow components of the NIR decay (Figure 3d). Following the same arguments as those surrounding eqs 1 and 2, we conclude that the green upconversion decay dynamics are “feeding-limited”: the decay rates of the feeding levels (^2^F_5/2_ and ^4^I_11/2_) are much slower than the decay rate of the emitting level, so the green upconversion decay is determined by the lifetime components of the feeding levels. The fast component of the green upconversion decay is due to direct feeding by the NIR levels and we expect a good fit to the model *k*_G_^fast^(*d*) = *k*_nr,G_^fast^ + 2*k*_r,NIR_^0^ρ(980 nm, *d*). Here, the prefactor 2 reflects that two NIR excitations produce one green excitation, as derived in eqs 1 and 2 (Figure 4f; red). We find that a lower prefactor but the correct LDOS dependence matches the data, i.e. *k*_G_^fast^(*d*) = *k*_nr,G_^fast^ + 0.57*k*_r,NIR_^0^ρ(980 nm, *d*). Again, the low prefactor is probably because of overlap with the slow decay component on the fitted time range, which makes our selection of the fast decay component difficult. The slow component of the green upconversion decay must be due to a fourth-order process, whereby the ^4^I_13/2_ IR level feeds the NIR levels, which subsequently feed the green emission. Indeed, here the slow component matches the model *k*_G_^slow^(*d*) = *k*_nr,G_^slow^ + 2.1*k*_IR,r_^0^ρ(1550 nm, *d*) with an LDOS dependence that confirms feeding from the ^4^I_13/2_ level and a prefactor clearly above 1. Consistent with our expectation, the steady-state population of the green-emitting level is low compared to that of the NIR and IR levels, so continued feeding dominates the decay dynamics after the excitation pulse is turned off. For red upconversion emission, we obtain similar results (Supporting Information Figure S12).

### Core–Shell versus Core-Only NCs

Our findings in Figures 2–4 show that the decay rates of the feeding levels in the NIR and IR determine the upconversion decay rate in core–shell NCs. This happens because the decay rate of the emitting levels is faster than that of the feeding levels (eq 2). Eq 2 also shows that in the opposite scenario, where the feeding levels decay faster than the emitting levels, the upconversion decay rate should be simply the decay rate of the emitting level itself. More precisely, the relevant comparison is between the decay-rate constant of the emitting level and 2× that of the feeding level, for a two-photon ETU process. We will now investigate the excited-state dynamics for red and green upconversion of core-only NCs, where coupling to high-energy surface vibrations decreases the excited-state lifetime of all relevant upconversion energy levels.^8,24,35^

**Figure 5:**
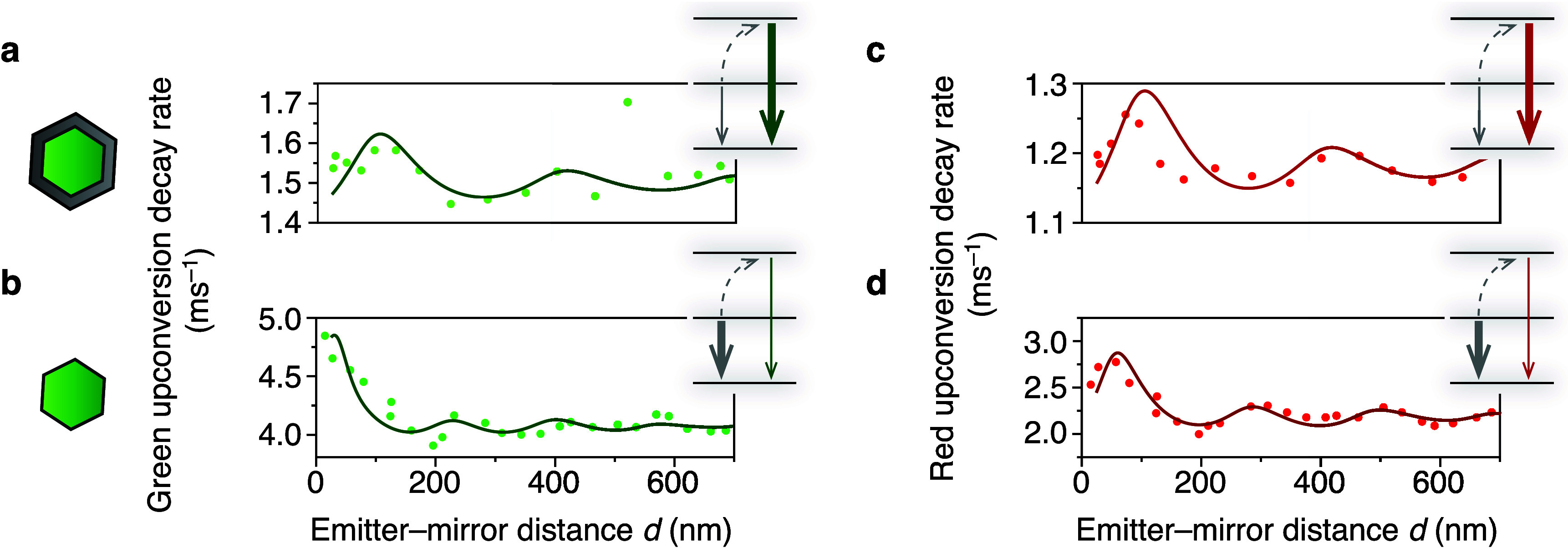
Rate-limiting step for green and red upconversion in core-only and core–shell NCs. (a) Green upconversion decay rate (fast component, same plot as Figure 4f) as a function of emitter–mirror distance for core–shell NCs with a fit to *k*_G_^fast^(*d*) = *k*_nr, G_^fast^ + 0.57*k*_r, NIR_^0^ρ(980 nm, *d*). Decay from the green-emitting level (thick green arrow) is faster than decay of the feeding level (thin gray arrow), which makes the upconversion dynamics feeding-limited. (b) Same as (a), but for core-only NCs. Here, both the feeding and emitting levels decay faster because of coupling to vibrations on the NC surface. The NIR-emitting levels are quenched more. As a result, upconverted emission from the green-emitting levels becomes lifetime-limited, and we observe a qualitatively different LDOS dependence. Solid line: fit to *k*_G_^fast^(*d*) = *k*_nr,G_^fast^ + *k*_r,G_^0^ρ(545 nm, *d*). (c) Red upconversion decay rate as a function of emitter–mirror distance for core–shell NCs showing feeding-limited upconversion dynamics. Solid line: fit to *k*_R_^fast^(*d*) = *k*_nr,R_^fast^ + *k*_r,R_^fast^ρ(980 nm, *d*), reflecting the feeding process. (d) Same as (c), but for core-only NCs. The lifetime of red emission after resonant excitation is hardly affected by the absence of a shell. We observe lifetime-limited upconversion dynamics. Solid line: fit to *k*_R_^fast^(*d*) = *k*_nr,R_^fast^ + *k*_r,R_^0^ρ(650 nm, *d*).

Figures 5a,b compare the green upconversion decay rate as a function of emitter–mirror distance for core–shell (Figure 5a) and core-only NCs (Figure 5b). Interestingly, we observe a qualitatively different modulation of the upconversion decay rate. The core–shell data match the LDOS at the wavelength of 980 nm of the feeding level, while the core-only data match the LDOS at the emission wavelength of 545 nm. We can understand this difference by identifying the faster decay rate. The green level *n*_G_ decays more quickly for the core–shell NCs, while (*n*_NIR_)^2^ decays more quickly for the core-only NCs. This inversion of the relative rates occurs because in core-only NCs the excited levels of dopants are quenched by coupling to surface-related vibrations, but this affects the feeding levels more strongly than the emitting levels. The cause must be the closer energy match of the ^4^I_11/2_–^4^I_13/2_ energy separation (3500 cm^–1^) with surface-adsorbed water^8,36^ compared to the ^4^S_3/2_–^4^F_9/2_ separation (3100 cm^–1^). As a result, decay from the green-emitting level is the lifetime-determining step for green upconversion emission in core-only NCs, while decay of the feeding levels is lifetime-determining in core–shell NCs. LDOS experiments (Figure 5a,b) visualize this qualitative difference directly. For red upconversion emission in core–shell and core-only NCs (Figure 5c,d), we observe exactly the same trend where the core-only data match the LDOS at the emission wavelength of 650 nm. Again, the slow components of red and green upconversion emission in core-only NCs both follow the 1550 nm LDOS (Supporting Information Figure S13), because the feeding by four-photon ETU processes are slower than direct decay from the emitting levels, as for the core–shell NCs (Figure 4 and Supporting Information Figure S12). These experiments demonstrate visually how interactions of the lanthanide dopants with molecules on the surface of the NC can change the upconversion pathways.

The insights provided into the upconversion rise and decay dynamics are relevant for various applications of upconverting materials. Some applications depend on the rise and decay dynamics directly. For example, upconversion materials have been proposed for background-free optical sensing based on the luminescence lifetime.^37−40^ In particular, temperature sensing based on the temperature dependence of the upconversion lifetime has been a topic of investigation. For accurate interpretation of sensing data, it will be crucial to account for the nontrivial dependence of the upconversion lifetime on the optical environment, excitation wavelength, excitation power (Figure 4c,e,f), and core–shell geometry (Figure 5). The rise and decay dynamics of upconversion are further relevant for background-free bioimaging, where upconversion lifetime could be used for multiplexing^41^ but slow rise and decay dynamics could limit imaging speeds.^42,43^ Other research directions optimize core–shell NC geometries and doping concentrations to boost upconversion intensities,^21,44−46^ or use dielectric or plasmonic structures for the same purpose.^47,48^ Our ramped-reflector measurements highlight the contribution of various energy-transfer pathways to upconversion and clearly show dependence of upconversion dynamics on the photonic environment. It is still an open question how these insights into upconversion dynamics can be used to maximize intensities. Follow-up work could focus on modeling of the steady-state emission intensities based on the excited-state decay pathways revealed in this work.

## Conclusions

To conclude, we have presented a detailed study on the rise and decay dynamics in NaYF_4_ upconversion NCs doped with Er^3+^and Yb^3+^. Both the rise and decay dynamics are nonexponential, but systematic photonic and power-dependent experiments allowed us to unravel different upconversion pathways. The rise depends on the excitation power and reveals the number of rate-determining steps in the process that populates the NIR- (1 step), green- (2 steps), and red-emitting (3 steps) levels. Using a photonic approach, we were able to identify the important ETU processes that lead to red and green upconverted emission. Systematically varying the photonic environments revealed how different decay components of the multiexponential decay are due to different feeding pathways. We could explain a crossover from decay-limited (core-only NCs) to feeding-limit (core–shell NCs) upconversion dynamics, highlighting how the NC geometry affect the upconversion dynamics qualitatively. Our insights will be important for materials development for applications that depend on upconversion dynamics directly as well as those that aim at brighter and more efficient upconversion.

## Experimental Methods

### Ramped-Reflector Substrate

Fiducial markers were etched into a Si wafer with direct laser writing. For this, a four-inch wafer was dehydrated for 5 min at 180 °C on a hot plate and treated with HDMS. The substrate was spin-coated with AZ1505 photoresist at 4000 rpm/2000 rpm s^–1^/40 s and post baked for 1 min at 110 °C on a hot plate. The fiducial markers were written into the photoresist with a direct-laser-writing tool (Heidelberg Instruments DWL 2000). After exposure, the resist was developed in a 1:4 AZ400 K:H_2_O solution for 20 s and rinsed with deionized water. The markers were transferred into the Si wafer with reactive-ion etching for ∼40 s, using 100 sccm Ar and 100 sccm SiF_6_ at a chamber pressure of 90 mTorr and an RF power of 40 W (Oxford Instruments PlasmaPro NPG 80). After etching, the markers were ∼330 nm deep. The wafer was cleaned by sonication in acetone followed by isopropyl alcohol for 3 min each, blowdried with 0.45-μm filtered nitrogen, and O_2_-plasma cleaned for 5 min at 600 W (PVA TePla GIGAbatch 310M). Prior to dicing, AZ1512 HS photoresist was spin-coated on the substrate at 4000 rpm/2000 rpm s^–1^/40 s and hot baked for 1 min at 110 °C to protect the wafer during the dicing process. The coated wafer was diced into 1 × 1 cm^2^ chips (ADT ProVectus LA 7100). Next, an optically thick Au film was evaporated on the Si chips. The substrates were cleaned by sonication in acetone followed by isopropyl alcohol, each for 3 min, and blowdried with 0.45-μm filtered nitrogen. The dried chips were O_2_-plasma cleaned for 5 min at 200 W (Diener Zepto). About 210 nm of Au was evaporated onto the substrate using a thermal evaporator (Kurt J. Lesker, Nano 36). The evaporation was done at a pressure of 1.22 × 10^–7^ mbar with a deposition rate of 1 nm s^–1^. Finally, the Al_2_O_3_ ramp was sputtered onto the Au-coated Si chips. Strips of about 1 mm in width were covered at opposite sides of the chip with clean glass slides to create two level planes. The rotating holder was mounted in the vacuum chamber of the magnetron sputterer (Kurt J. Lesker PVD 75). The chip was covered by a custom-made metallic shadow mask, which was placed a few millimeters from the chip’s surface. After the chamber was pumped down overnight, Al_2_O_3_ was deposited in a reactive-sputtering process from an Al target using a partial injection of 20 vol % O_2_ in Ar. Sputtering was performed at a pressure of 1 mTorr, power of 200 W, reflected power of <5 W, and initial DC bias of ∼160 V, yielding a deposition rate of 5 nm min^–1^. During deposition, the holder was rotated from underneath the shadow mask—to gradually expose fresh Au surface to the Al_2_O_3_ flux—in 14 steps of 10 min with increments of 0.7°.

### Ramped-Reflector Measurements

Diluted dispersions (∼5 mg mL^–1^) were spin-coated on the ramped-reflector substrates to achieve submonolayer coverage.

#### Upconversion Excitation

A 980-nm laser (OBIS LX 980 nm) was guided to the sample by a 50/50 beamsplitter (Thorlabs, BSW26R) and focused by a 40× Nikon CFI Plan Fluor (NA = 0.75) air objective on the ramped-reflector substrate. The laser was operated in pulsed mode, externally driven by a square-wave voltage profile using a TTi TGA1244 function generator. The green upconverted ^2^H_11/2_ → ^4^I_15/2_ emission line was selected using band-pass filters (Chroma ET535/70M and Chroma ET519/26M), to reject the ^2^H_9/2_ emission which overlaps with the ^4^S_3/2_ → ^4^I_15/2_ emission. The red upconverted ^4^F_9/2_ → ^4^I_15/2_ emission line was selected using band-pass filters (Thorlabs FELH650 and FESH700), the FESH700 filter was placed under an angle to blueshift the onset of the transmission/absorption edge toward the 650-nm emission. All lanthanide emission was collected by the same objective and collimated outside of the microscope using a relay lens system and subsequently focused on silicon avalanche photodiodes (APDs; MPD PDM or Thorlabs SPDMH2), which sets the maximum detected wavelength to approximately 1100 nm, further restricted by the filters that we use in the different experiments. The laser pulses and photon-detection events were synchronized using a quTools QuTAG time-to-digital convertor and processed using custom software for data storage.

#### Resonant Excitation

For the resonant-excitation measurements, the same optical path was used. However, different lasers for NIR (OBIS LX 980 nm), red (OBIS LX 637 nm), and green (OBIS LX 522 nm) emission were used. For the NIR (FESH100) and green emission (ET519/26M), additional cleanup filters in the excitation path were used to reject the low-energy tail of the laser line. The resonant emission line was selected using appropriate filters for the NIR (FELH100), red (FELH650 and FESH700), and green (ET546/10x) emission line.

### Power-Dependent Rise and Decay Measurements

A stock solution was dropcasted on a glass coverslip to obtain thick films of core-only and core–shell NCs. A high-magnification oil-immersion objective (Nikon CFI Plan Apochromat Lambda 100×, NA = 1.45) was used to achieve a small laser spot size and a wide range of excitation fluences. The excitation fluence on the sample was adjusted by a motorized filter wheel (FW102C) in the excitation path, equipped with neutral-density filters with optical densities (ODs) between 0 and 4. To avoid damaging our APDs, we adjusted the signal rate using a similar motorized filter wheel (FW212C) in the emission path. The OD values at the relevant emission/excitation wavelengths were measured by placing the filters in an absorbance spectrograph and were used to retrieve the relative emission rates vs excitation fluence. We measured the spot size by imaging the reflection of the 980 nm laser off a glass–air interface on a Andor iXon 888 EMCCD and found a spot size of *r* = 1.3 μm (standard deviation of Gaussian fit). The power on the sample was measured using a Thorlabs power meter (PM100D) equipped with a Si photodiode (S170C). From the spot area *A* = π*r*^2^, excitation power *P*, photon energy *E*, and absorption cross section of Yb^3+^ (σ = 7.8 × 10^–21^ cm^2^ from ref (49), we calculate the excitation rate using *k*_ex_ = *P*σ/*AE*.

## Supporting Information Available

Electron-microscopy characterization of upconversion nanocrystals, deadtime correction procedure, self-interference model, extended data of power-dependent rise/decay and photon experiments on core-only and core–shell nanocrystals (PDF)
