# Supplementary material for: Rise and Decay of Photoluminescence in Upconverting Lanthanide-Doped Nanocrystals
Source: ACS Nano. 2024 Oct 5;18(41):28325–34. doi: 10.1021/acsnano.4c09945 (PMC11483940; doi:10.1021/acsnano.4c09945)
Supplement: Supplementary file 1 — nn4c09945_si_001.pdf [file nn4c09945_si_001.pdf]

Supporting Information for

Rise and decay of photoluminescence in upconverting lanthanide-doped nanocrystals

Sander J.W. Vonk[1,2]*, J.J. Erik Maris*[4]*, Ayla J.H. Dekker*[1,3]*, Jur W. de Wit*[1]*, Thomas P. van Swieten*[1]*, Ario Cocina*[4] *& Freddy T. Rabouw**[1,2]

[1]Soft Condensed Matter & Biophysics, Debye Institute for Nanomaterials Science, Utrecht University, Princetonplein 1, 3584 CC Utrecht, The Netherlands

[2]Inorganic Chemistry & Catalysis, Debye Institute for Nanomaterials Science & Institute for Sustainable and Circular Chemistry, Utrecht University, Universiteitsweg 99, 3584 CG Utrecht, The Netherlands

[3]Organic Chemistry & Catalysis, Institute for Sustainable and Circular Chemistry, Utrecht University, Universiteitsweg 99, 3584 CG Utrecht, The Netherlands

[4]Optical Materials Engineering Laboratory, ETH Zürich, Leonhardstrasse 21, 8092 Zürich, Switzerland

* Corresponding Author: f.t.rabouw@uu.nl

S1 Characterization of upconverting nanocrystals

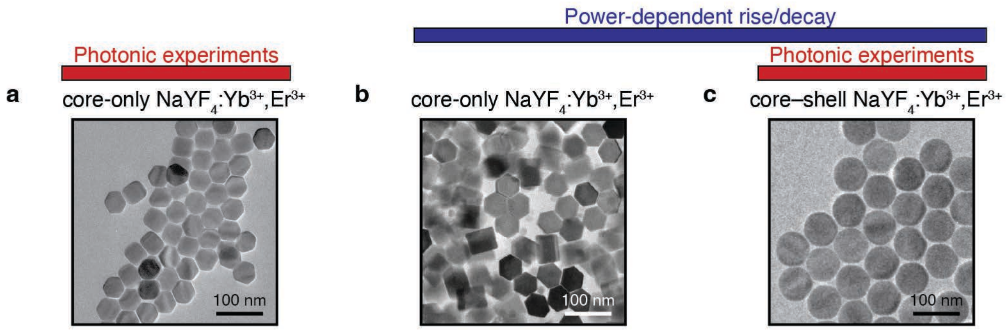

Figure S1 | Characterization of upconverting NCs. (a) Electron-microscopy image of $NaYF_4$:Er^{3+},Yb^{3+} NCs. The NCs have a diameter of 46.3 ± 3.3 nm (mean $\pm$ standard deviation, averaged over 100 NCs). **(b)** Same as **a**, but for a different synthesis batch of core-only NCs. The NCs have a diameter of 49.8 ± 4.4 nm (mean $\pm$ standard deviation, averaged over 100 NCs). **(c)** Same as **a**,**b**, but for core–shell NCs synthesized using the cores in **b**. The NCs have a diameter of 72.8 ± 3.9 nm (mean $\pm$ standard deviation, averaged over 100 NCs). All electron-microscopy images were acquired with a Tecnai 20 transmission-electron microscope.

S2 Rise and decay for 2-level systems

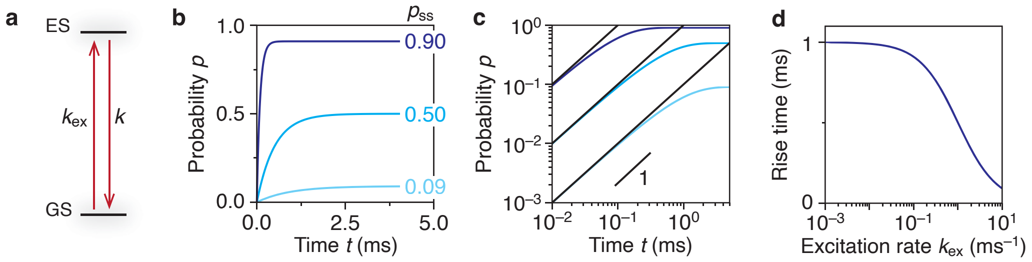

Figure S2 | Rise time of 2-level systems. (a) The ground state (GS) and excited state (ES) of a 2-level system are coupled by excitation with rate constant k_{ex} (upward arrow) and decay with rate constant k (downward arrow). **(b)** Probability p that a 2-level system with $k = 1$ ms^{-1} is in the ES after turning on the excitation at $t = 0$ for different $k_{ex} = \{0.1, 1, 10\}$ ms^{-1} (light blue to dark blue). The steady-state probability p_{ss}, for $t \to \infty$, increases with excitation rate. **(c)** Same as **b**, but plotted on a log–log scale. The initial rise is linear for all excitation rates (black lines) and the initial slope increases linearly with k_{ex}. **(d)** Rise time as a function of excitation rate following $\tau_{rise} = (k + k_{ex})^{-1}$ (solid blue line).

In a 2-level system, absorption of light (with excitation rate k_{ex}) and excited-state decay (decay rate k) couple the excited state ES and the ground state GS (Fig. S2). Here, the total decay rate $k = k_r + k_{nr}$ consists of radiative k_r and nonradiative k_{nr} decay. The differential equation, describing the change in probability in time t that an ion is in the excited state p, is given by:

$$\frac{dp}{dt} = -kp + k_{ex}(1 - p). \tag{1}$$

We can solve this equation analytically for the rise after turning on the excitation after $t = 0$ [*i.e.* $p_{rise}(t = 0) = 0$] yielding

$$p_{rise}(t) = p_{ss}[1 - e^{-(k+k_{ex})t}], \tag{2}$$

where $p_{ss} = k_{ex}(k + k_{ex})^{-1}$ is the steady-state probability for $t \to \infty$. In a real experiment, we excite a large number of ions N with radiative decay rate k_r, which yields the photon-emission rate ϕ given by

$\phi = Nk_r p$. Fig. S2b plots Eq. 2 for $k = 1$ ms^{-1} and varying excitation rates $k_{ex} = \{0.1, 1, 10\}$ ms^{-1} (light blue to dark blue). We observe an in-growth of p_{rise} to the steady-state population p_{ss}. The slope of the initial in-growth in time and the steady-state population p_{ss} both increase with the excitation rate. The same plots, but on a log–log scale (Fig. S2c), reveal that the initial in-growth is linear for all excitation rates. We can understand this by expanding Eq. 2 up to first order in t around $t = 0$:

$$p_{rise}(t) = k_{ex}t + \mathcal{O}(t^2), \tag{3}$$

which indeed shows that the initial in-growth goes linearly in time and that the slope of the initial rise increases linearly with the excitation rate. From Eq. 2, we directly see that the rise time, *i.e.* the time to reach $(1-1/e)p_{ss}$, is equal to $\tau_{rise} = (k + k_{ex})^{-1}$. The rise time as a function of excitation rate (Fig. S2d) shows a dependence qualitatively similar to the saturation measurement of the Mn^{4+}-based phosphor presented in Fig. 1 of the main text.

The decay of the excited-state population after the laser is turned off is straightforwardly obtained from the differential equation in Eq. 1 for $k_{ex} = 0$ ms^{-1}. For a population p_0 directly after the laser is turned off, we obtain

$$p_{decay}(t) = p_0 e^{-kt}, \tag{4}$$

which shows simple power-independent single-exponential decay with slope k.

S3 Correcting missed photon-detection events because of the detector deadtime

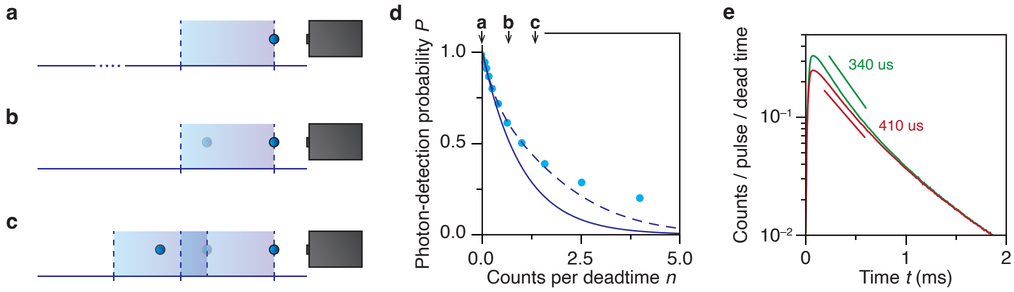

Figure S3 | Correcting for the detector deadtime at high signal rates. **(a)** A photon-detection event by an avalanche photodiode (APD) triggers the detector deadtime period of length δt, during which the detector is unable to detect any further incoming photons. For small signal rates, no photons are missed as the probability that a photon falls within a previous photon's deadtime is negligible. **(b)** At higher signal rates, incoming photons are missed because of the detector deadtime. The earliest photon (blue circle, on the right) arrives at the detector and triggers a deadtime period (blue-shaded region), so that the second photon (transparent blue circle, on the left) is not recorded. **(c)** At even higher signal rates, it is difficult to predict the fraction of the photons that are missed because of the deadtime. The cartoon shows a photon-triplet event. The latest photon (on the left) arrives on the detector within δt of the second photon (in the middle). The latest photon is nevertheless recorded successfully because the second photon was missed as it hits the detector within the deadtime period following the earliest photon. **(d)** Monte-Carlo-simulated photon-detection probability P as a function of signal rate in units of average counts n per deadtime. Solid blue: Eq. 5, photon-detection probability accounting for missed photons after a triggered deadtime. Dashed line: Eq. 6, photon-detection probability corrected for photon-triplet events described in **c**. At higher signal rates, we fail to reproduce the simulated results because of photon quadruplets and higher multiplets, but these are irrelevant at the signal rates we used in our experiments. **(e)** Red: Measured decay curve of green upconversion emission ($k_{ex} = 0.4$ ms^{-1} and 50-μs pulse duration) of core–shell upconverting NCs doped with Yb^{3+} and Er^{3+}. At the decay-curve maximum the recorded count rate is $n_{out} \approx 0.1$ counts per deadtime, so the response of the detector is nonlinear (*i.e.*, P is significantly different from 1). Green: Reconstructed input signal rate on the detector n_{in}. We determine n_{in} by solving $n_{in}P(n_{in}) = n_{out}$. The fitted fast lifetime component (fitted on $t = 0.1$–0.4 ms) is different by 20%, depending on whether we analyze the recorded

signal (red) or the reconstructed input signal (green).

In our measurements of the excited-state decay, we use a Si-based avalanche photodiode (APD) to record single photons with high timing precision. To achieve a high dynamic range—*i.e.* decay over many orders of magnitude—we need a high signal rate on top of the background rate due to *e.g.* dark counts. At high signal rates, the detectors may fail to detect photons because of the detector deadtime: the detector is unable to record any additional photons for a period of duration δt after a detection event (Fig. S3a, blue shaded area). δt is typically several tens of nanoseconds for APDs and photomultiplier tubes. In Fig. S3d, we investigate the effect of the detector deadtime with a Monte Carlo simulation of the photon-detection probability (fraction of photons that do *not* fall within an earlier photon's deadtime period) for varying average number of input counts $n \in [0, 5]$ per deadtime (blue dots). To reproduce the simulated results analytically, we calculate the probability P_b that *no* photons hit the detector within any period of duration δt:

$$P_\mathrm{b} = \frac{n}{\delta t} \int_{\delta t}^{\infty} \mathrm{e}^{-nt/\delta t} \mathrm{d}t = \mathrm{e}^{-n}, \tag{5}$$

where the exponential distribution $k\mathrm{e}^{-kt}$ is the waiting-time distribution for signal rate $k = n/\delta t$. We plot P_b in Fig. S3d (blue solid line), which reproduces the photon-detection probability P for $n < 0.2$.

At high count rates, multiple photons may hit the detector on the timescale of the deadtime. From the simulations, we observe that Eq. 5 underestimates the photon-detection probability (Fig. S3d). In that regime, triplet events such as schematically depicted in Fig. S3c appear. We calculate the probability P_c of such an event:

$$P_\mathrm{c} = \left(\frac{n}{\delta t}\right)^2 \int_0^{\delta t} \int_{t'}^{t'+\delta t} \mathrm{e}^{-nt'/\delta t} \mathrm{e}^{-n(t''-t')/\delta t} \mathrm{d}t'' \mathrm{d}t' = \mathrm{e}^{-2n}[1 + \mathrm{e}^{n}(n-1)], \tag{6}$$

where t'' is the delay time between the earliest photon and the latest photon of the photon triplet (see Fig. S3c) and t' is the delay time between the earliest and the second photon. We correct the photon-detection probability by adding these events to Eq. 5 and find

$$P_\mathrm{b+c} = \mathrm{e}^{-2n} + \mathrm{e}^{-2n}[1 + \mathrm{e}^{n}(n-1)]. \tag{7}$$

We observe that we correctly model the photon-detection probability up to an input signal rate of $n = 1$ per deadtime (Fig. S3), which is well above the signal rates in our experiments. In principle, even higher-order events than the triplets of Fig. S3c can be included in the analytical model to match the simulated results for higher n. In our experiments at modest signal rates of $n < 1$, we choose to use Eq. 7 to reconstruct the input signal rate n_in from the recorded signal rate n_out by inverting $n_\mathrm{in} P_\mathrm{b+c}(n_\mathrm{in}) = n_\mathrm{out}$.

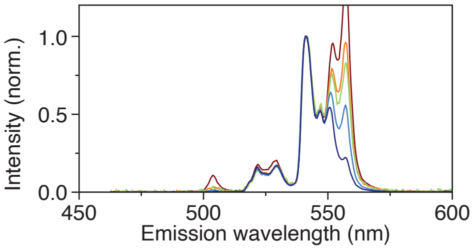

Figure S4 | Negligible laser heating in power-dependent decay-curve measurements. Upconverted emission spectra of core-only $NaYF_4$:Yb^{3+},Er^{3+} NCs upon 980-nm excitation, normalized to the $^4S_{3/2} \rightarrow {}^4I_{15/2}$ emission. Here, we used the exact same excitation rates k_ex as for the power-dependent measurements in Fig. 2 of the main text. The color scale of the graphs visually depicts the magnitude of the excitation rate from slow (blue) to fast (red). The stable intensity ratio between the $^4S_{3/2} \rightarrow {}^4I_{15/2}$ (520 nm) and $^2H_{11/2} \rightarrow {}^4I_{15/2}$ (540 nm) emissions as a function of excitation rate shows that self-heating by 980-nm absorption is negligible. At the highest excitation rate (dark red) however, the intensity ratio is slightly distorted which might reflect self-heating or overlapping higher-order upconversion emission with $^2H_{11/2} \rightarrow {}^4I_{15/2}$ emission.

S4 Modeling the decay rate from a self-interference model

To model the decay rate of the lanthanide-doped NCs in our experiments, we model our NCs as emitters of mixed isotropic electric-dipole (ED) and magnetic-dipole (MD) emission[S1] embedded in a three-layer mirror geometry (Fig. 3 of the main text). Any preferential transition-dipole orientation or shape-induced polarized emission is neglected, because our nanocrystals are approximately spherical (Fig. S1) and likely lie on the photonic substrate with random orientations. In our model, layer 0 is the top air layer ($\epsilon_0 = 1$) containing the emitters, layer 1 is the Al_2O_3 spacer layer, and layer 2 is the gold reflector with wavelength-dependent dielectric function[S3]. We obtained the best match with experiments if we took slightly different permittivity values for the Al_2O_3 spacer between the substrates used for the core–shell sample ($\epsilon_1 = 3$) compared to the core-only sample ($\epsilon_1 = 2.7$), probably because of stoichiometry differences in the Al_2O_3. In this mirror geometry, the polarization-averaged local density of optical states (LDOS) for ED and MD transitions is given by[S2]:

$$\rho^{\mathrm{ED}} = \int_0^{u_\mathrm{m}} \Big\{ \Big[1 + \Big(\frac{1}{2} r_\mathrm{s} + \frac{1}{2} r_\mathrm{p} - u^2 r_\mathrm{p} \Big) \mathrm{e}^{-2 l_0 k_0 h} \Big] \frac{u}{l_0} \Big\} \mathrm{d}u, \tag{8}$$

$$\rho^{\mathrm{MD}} = \int_0^{u_\mathrm{m}} \Big\{ \Big[1 - \Big(\frac{1}{2} r_\mathrm{s} + \frac{1}{2} r_\mathrm{p} - u^2 r_\mathrm{p} \Big) \mathrm{e}^{-2 l_0 k_0 h} \Big] \frac{u}{l_0} \Big\} \mathrm{d}u, \tag{9}$$

where $k_0 = 2\pi/\lambda$ is the vacuum wave vector of emitted (or absorbed) light, $l_j = -\mathrm{i}\sqrt{\epsilon_j/\epsilon_1 - u^2}$, h is the distance between the emitter and the Al_2O_3/air interface, and $u = k_\parallel/k$ the fractional in-plane momentum of electromagnetic radiation. From this expression, we can calculate the total LDOS ρ using $u_\mathrm{m} = \infty$ and the excitation enhancement ρ_ex using $u_\mathrm{m} = \mathrm{NA}/\sqrt{\epsilon_0}$, where NA is the numerical aperture of the microscope objective. The LDOS depends on the spacer thickness d via the three-layer Fresnel coefficients $r_{\mathrm{s,p}}$, given by

$$r_{\mathrm{s,p}} = \frac{r^{01}_{\mathrm{s,p}} + r^{12}_{\mathrm{s,p}} \mathrm{e}^{-2 l_1 k_0 d}}{1 + r^{01}_{\mathrm{s,p}} r^{12}_{\mathrm{s,p}} \mathrm{e}^{-2 l_1 k_0 d}}, \tag{10}$$

where $r^{01}_{\mathrm{s,p}}$ and $r^{12}_{\mathrm{s,p}}$ are the Fresnel coefficients for reflection of s-and p-polarized light on the interface between layers 0/1 and 1/2, respectively. The Fresnel coefficient for the interface between medium i and j is given in terms of the dielectric constants ϵ_i and the emission direction u by

$$r^{ij}_\mathrm{s} = \frac{l_i - l_j}{l_i + l_j}, r^{ij}_\mathrm{p} = \frac{\epsilon_j l_i - \epsilon_i l_j}{\epsilon_j l_i + \epsilon_i l_j}. \tag{11}$$

We use the total LDOS at different emission wavelengths to model the total decay rate k of the green, red, NIR, and IR emitting levels of the Er^{3+}/Yb^{3+} couple:

$$k = k_\mathrm{nr} + a k_\mathrm{r}^{\mathrm{bulk}} \sum_i \Big[\frac{1}{n_\mathrm{NC}} \chi^2_\mathrm{ED} f_i^\mathrm{ED} \rho^\mathrm{ED}(\lambda_i) + \Big(\frac{1}{n_\mathrm{NC}} \Big)^3 \chi^2_\mathrm{MD} f_i^\mathrm{MD} \rho^\mathrm{MD}(\lambda_i) \Big], \tag{12}$$

where k_nr is the nonradiative decay rate, $k_\mathrm{r}^\mathrm{bulk}$ is the radiative decay rate in bulk $NaYF_4$, $n_\mathrm{NC} = 1.48$ is the refractive index of the $NaYF_4$ NCs[S4], $\chi_\mathrm{ED} = 3/(2 + n^2_\mathrm{NC})$ and $\chi_\mathrm{MD} = 1$ are the local-field factors of a NC in vacuum, and $f_i^{\mathrm{ED/MD}}$ are the branching ratios for the ED/MD transitions at emission wavelength λ_i from the same excited state. The bulk radiative decay rates[S5] and branching ratios and ED/MD contributions[S6] for the relevant emitting levels are summarized in Table S1.

Table S1: Branching ratios of the green, red, NIR, and IR transitions of Yb^{3+} and Er^{3+}in $NaYF_4$.

	$k_\mathrm{r}^\mathrm{bulk}$ (ms^{-1}) [S5]	trans. 1	f_1^ED	f_1^MD [S1]	trans. 2 [S6]	f_2^ED
G	1.55	$^2H_{11/2}$+$^4S_{3/2} \rightarrow {}^4I_{15/2}$ (545 nm)	0.79	N/A	$^2H_{11/2}$+$^4S_{3/2} \rightarrow {}^4I_{13/2}$ (850 nm)	0.21
R	1.90	$^4F_{9/2} \rightarrow {}^4I_{15/2}$ (650 nm)	0.84	N/A	$^4F_{9/2} \rightarrow {}^4I_{13/2}$ (1120 nm)	0.16
NIR	0.58	$^2F_{5/2} \rightarrow {}^2F_{7/2}$ (980 nm)	0.91	0.09	N/A	N/A
IR	0.13	$^4I_{13/2} \rightarrow {}^4I_{15/2}$ (1500 nm)	0.74	0.26	N/A	N/A

In the main text, we fit Eq. 12 to decay components of decay curves from the ramped-reflector measurements using a least-squares procedure to fit the nonradiative decay rate k_nr and the bulk scaling factor a. We also allow the fit procedure to find a deviation c_i in the emitter–mirror distance from the calibrated value d_i for each data point. This strategy accounts for uncertainties in emitter–mirror distance but also for uncertainties in Al_xO_y stoichiometry, which affect the phase advance as light travels through the spacer layer. We make sure that the

algorithm finds reasonable values of c_i with a regularization term in the error function:

$$E(k_{\mathrm{nr}}, a) = \sum_{i}^{\text{data points}} [k_i^{\exp} - k(d_i + c_i)]^2 + f c_i^2. \quad (13)$$

Here $k_i^{\exp}$ is an experimental decay rate, $k(d_i + c_i)$ is Eq. 12 at an emitter–mirror distance of $d = d_i + c_i$, and f is a constant that ensures reasonable optimal values of c_i. We found that a good value is typically $f = 2 \times 10^{-4}$–2×10^{-7} $\mathrm{ms}^2\,\mathrm{nm}^{-2}$, which yields values for c_i of on average 5–15 nm. For all experiments in the main text, we test which LDOS scaling from Table S1 provides the best match by comparing the minimum values of E. The best matching LDOS scaling, fitted radiative and nonradiative rates of all ramped-reflector measurements are summarized in Table S2 and Table S3.

Table S2: Fitted nonradiative and radiative decay rates in core–shell upconverting NCs.

Transition	Excitation	Component	ρ	k_{nr} (ms^{-1})	a
NIR	resonant	intermediate	980 nm	0.332	0.607
	resonant	slow	1550 nm	0.152	1.594
G	resonant	N/A	545 nm	2.021	1.092
	upconversion	fast	980 nm	1.315	0.572
	upconversion	slow	1550 nm	0.281	2.077
R	resonant	N/A	650 nm	1.350	0.741
	upconversion	fast	980 nm	1.019	0.504
	upconversion	slow	1550 nm	0.305	1.564

Table S3: Fitted nonradiative and radiative decay rates in core-only upconverting NCs.

Transition	Excitation	Component	ρ	k_{nr} (ms^{-1})	a
NIR	resonant	slow	1550 nm	0.270	2.039
G	resonant	N/A	545 nm	2.933	1.312
	upconversion	fast	545 nm	3.580	0.590
	upconversion	slow	1550 nm	0.755	8.973
R	resonant	N/A	650 nm	0.860	1.217
	upconversion	fast	650 nm	1.440	0.717
	upconversion	slow	1550 nm	0.773	9.627

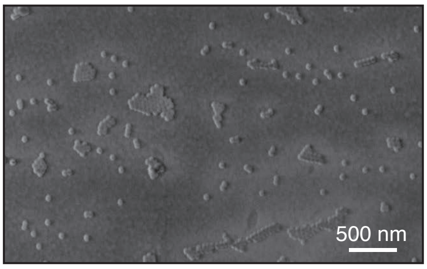

Figure S5 | Scanning electron-microscopy image of the ramped-reflector substrate coated with core-only $NaYF_4$:Yb^{3+},Er^{3+} NCs showing submonolayer coverage.

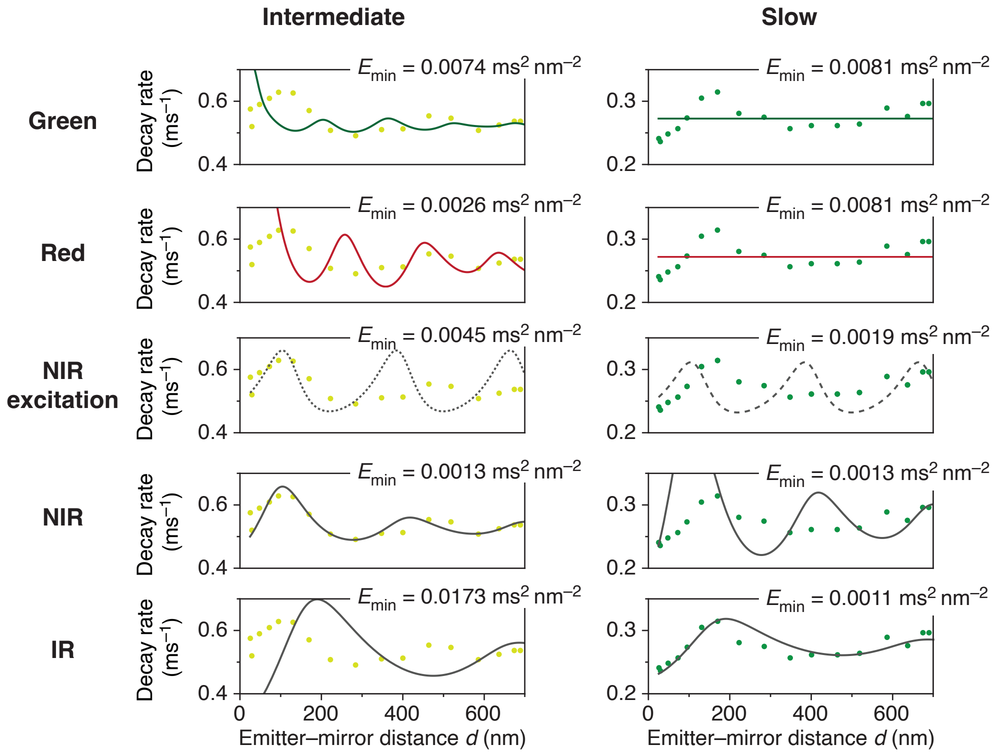

Figure S6 | Attempted LDOS fits to the intermediate and slow NIR decay components. Fit results of our LDOS model (Eq. S8–12) to the intermediate (left panels) and slow (right panels) decay components as a function of emitter–mirror distance for NIR emission of Er^{3+} and Yb^{3+}. We minimize the error function eq. S13 for green (545 nm), red (650 nm), NIR (980 nm), IR (1550 nm) emission and 980-nm NIR excitation to find the best matching LDOS dependence. The fits with the smallest sum of squared residuals (RSS) for the intermediate (980-nm LDOS) and slow (1550-nm LDOS) decay components are depicted in Fig. 3 of the main text.

S5 Initial rise for multi-step feeding

Here, we will derive that the power exponent of the initial rise is equal to the number of transition processes required to go from the ground state to the emitting state (Fig. S7a). We consider the general situation where the transition from the ground state 0 to the emitting state n occurs through a series of intermediate states $\{1, 2, \ldots, n-1\}$, coupled with rate constants $k_{i,i+1}$ with $i \in \{0, 1, \ldots, n-1\}$.

For short times after the start of the excitation pulse, the population in the ground state remains unity: $p_0 \approx 1$. The population of state 1 is described by

$$\frac{\mathrm{d}p_1}{\mathrm{d}t} = k_{01} p_0 \approx k_{01}. \tag{14}$$

With the approximation of a constant $p_0 \approx 1$ and an initial condition of $p_1(0) = 0$, the solution to this differential equation is $p_1 = k_{01}t$. Subsequently, p_1 feeds p_2:

$$\frac{\mathrm{d}p_2}{\mathrm{d}t} = k_{12} p_1 \approx k_{12} k_{01} t. \tag{15}$$

With the boundary condition $p_2(0) = 0$, the solution is $p_2 = \frac{1}{2} k_{01} k_{12} t^2$. After n steps, we obtain for the population of the emitting state that

$$p_n(t) = \frac{t^n}{n!} \prod_{i=0}^{n-1} k_{i,i+1}. \tag{16}$$

This shows that the power-law slope n of a rise curve equals the number of steps and the prefactor is proportional to the product sum of all rate constants $k_{i,i+1}$.

We plot the initial rise of p_5, fed by a 5-step process (Fig. S7b,c). This is the expected number of steps preceding the population of the green-emitting level: absorption of two photons, ET from Yb^{3+} to ground-state Er^{3+}, ETU from Yb^{3+} to the NIR excited state of Er^{3+}, and MPR. The plot is obtained numerically integrating rate equations without the approximation of a constant $p_0 \approx 0$. Fig. S7c shows $p_5(t)$ if all steps have rate constants of order 10 ms^{-1}. We observe that the rise is proportional to t^5 (Fig. S7c, black line) as expected from Eq. 16. Next, we increase the rate constants for 3 out of the 5 processes by 2 orders of magnitude (Fig. S7d) to a value of $k_{\text{fast}} = 10^3$ ms^{-1}. The initial rise still goes with t^5. However, the slope changes to a value of 2 at time $t = 1/k_{\text{fast}}$. In our experiments we probe time and intensity scales highlighted by the black square, and observe the initial rise appears quadratic t^2. The observed power exponent of 2 reveals that two transition steps are rate-limiting on the timescales of our experiment. For red and green upconversion, these must be the two photon absorption events by Yb^{3+}, which have associated rate constants of $k_{\text{ex}} = 10^{-3} - 10^1$ ms^{-1}, slower than the time resolution of our experiments.

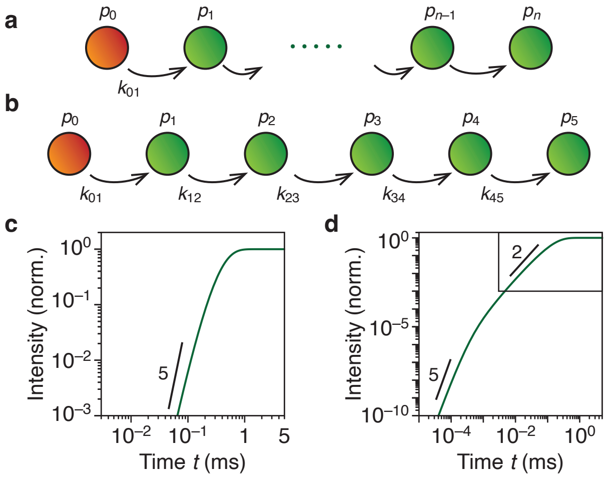

Figure S7 | Initial rise of multi-step feeding. (a) Schematic depiction of a n-step chain of feeding processes. **(b)** Schematic depiction of a 5-step chain of feeding processes, equivalent to the number of steps for green upconversion. **(c)** Rise of p_5, if all rate constant are of order 10 ms^{-1}. **(d)** Same as **c**, but here 3 out of the 5 steps have a rate constant of $k_{\mathrm{fast}} = 10^3\ \mathrm{ms}^{-1}$, boosted by a factor 100. The power-law slope of the rise dynamics changes from 5 to 2 when t approaches $t = 1/k_{\mathrm{fast}}$. The initial rise appears quadratic on the accessible time- and intensity range in our experiment (black rectangle).

S6 Extended data

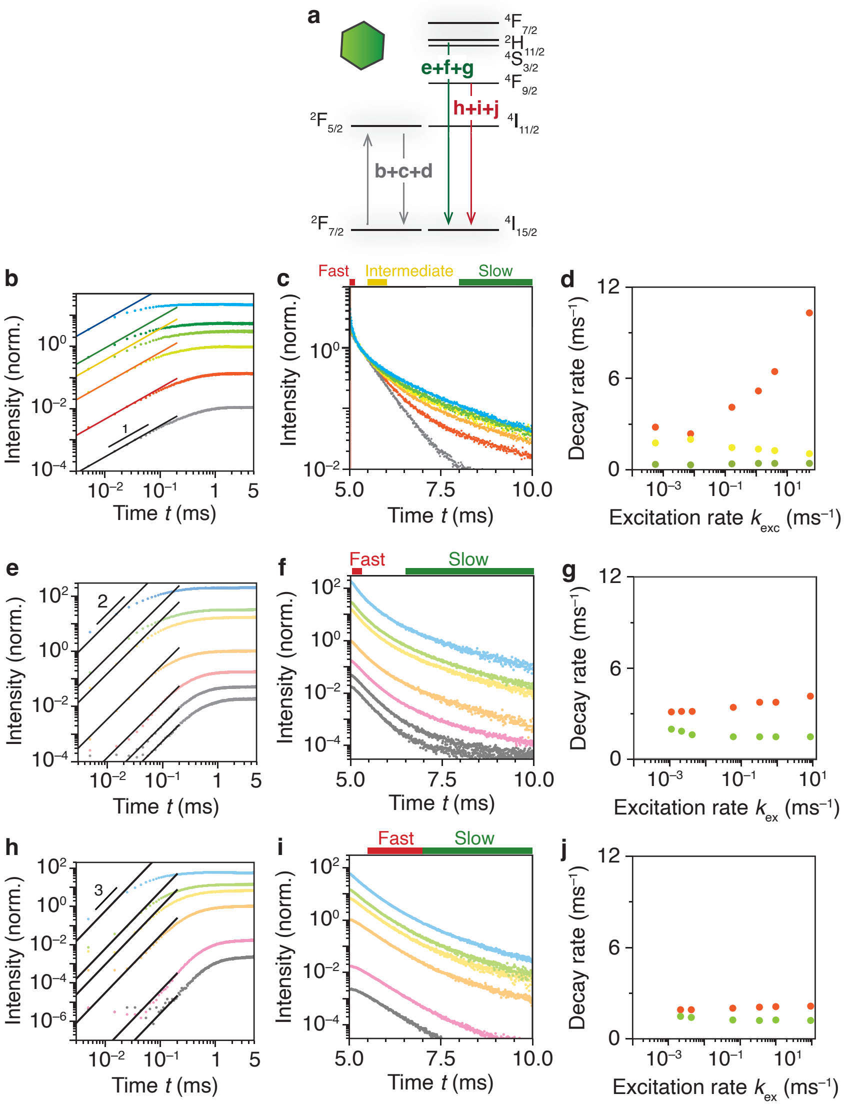

Figure S8 | Power-dependent rise and decay of upconverted emission in core-only NCs. **(a)** 980-nm excitation gives rise to NIR (**b,c,d**), green (**e,f,g**), and red emission (**h,i,j**). **(b)**–**(d)** Power-dependent NIR rise **b**, decay **c**, and extracted decay rates **d** of the fast (red), intermediate (yellow), and slow component (green). The decay curves are normalized to $t = 5.3$ ms to visualize the appearance of additional fast and slow components at higher excitation powers. **(e)**–**(g)** Same as **b**–**d**, but for the green upconverted emission. The rise and decay curves are normalized to the steady-state intensity of the experiment at $k_{ex} = 0.07\ \text{ms}^{-1}$. **(h)**–**(j)** Same as **b**–**d**, but for the red upconverted emission.

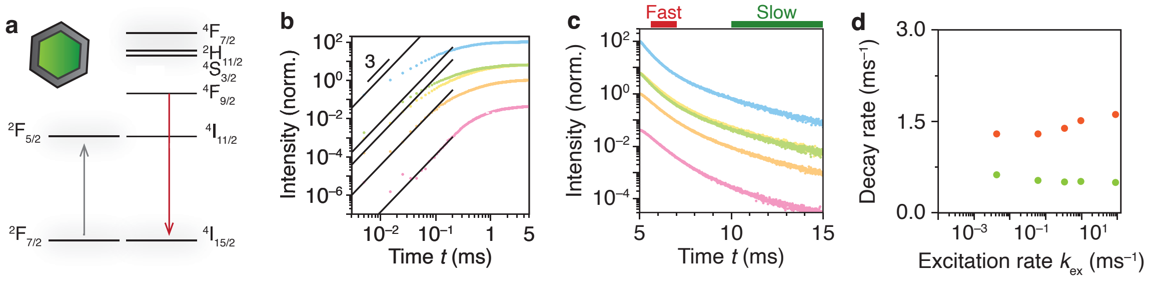

Figure S9 | Power-dependent rise and decay of red upconverted emission in core–shell NCs. (a) 980-nm excitation gives rise to red upconverted emission. **(b)–(d)** Power-dependent red upconverted rise **b**, decay **c**, and extracted decay rates **d** of the fast (red), and slow component (green). The rise and decay curves are normalized to the steady-state intensity of the experiment at $k_{ex} = 0.07$ ms^{-1}.

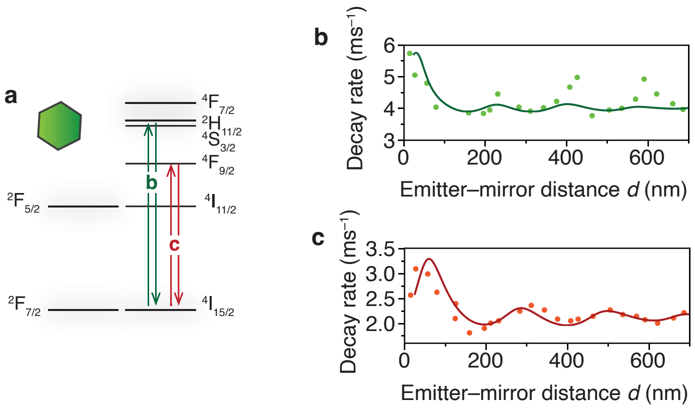

Figure S10 | Photonic experiments of red and green emission upon resonant excitation for core-only NCs. (a) Resonant 520-nm excitation gives rise to green emission and 637-nm excitation to red emission. **(b)** Extracted decay rate of green emission as a function of emitter–mirror distance. The LDOS at 545 nm modulates the decay rate, which is the emission wavelength of the electronic transition. **(c)** Same as **b**, but for red emission. The decay rate is modulated by the LDOS at 650 nm.

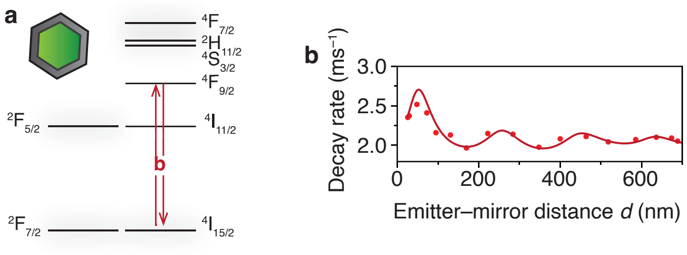

Figure S11 | Photonic experiments of red emission upon resonant excitation for core–shell NCs. (a) Resonant 637-nm excitation gives rise to red emission. **(b)** Extracted decay rate of red emission as a function of emitter–mirror distance. The decay rate is modulated by the LDOS at 650 nm, the emission wavelength of the electronic transition.

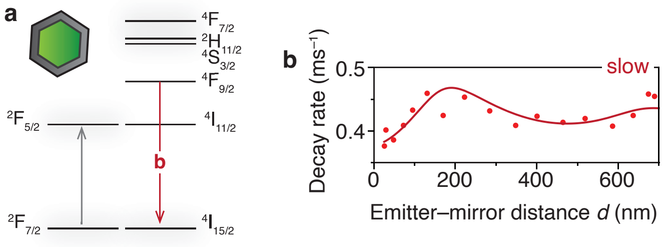

Figure S12 | Photonic experiments of red upconverted emission in core–shell NCs. (a) 980-nm excitation gives rise to red upconverted emission. **(b)** Extracted decay rate of red emission as a function of emitter–mirror distance. The decay rate is modulated by the LDOS at 1550 nm, the emission wavelength of the $^4I_{13/2}$ feeding level at these timescales.

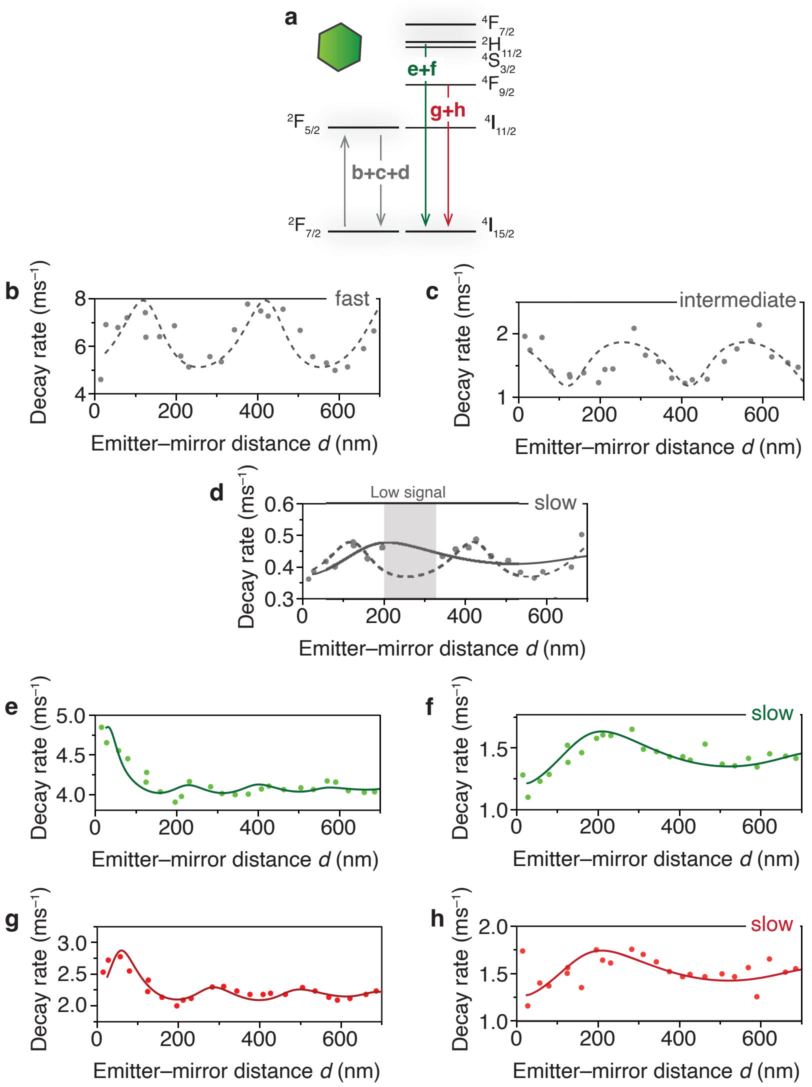

Figure S13 | Photonic experiments of NIR, green, and red emission for core-only NCs. **(a)** 980-nm excitation gives rise to NIR (**b**,**c**,**d**), green (**e**,**f**), and red emission (**g**,**h**). **(b)**–**(d)** Fitted decay rates of the fast **b**, intermediate **c**, and slow **d** NIR decay component as a function of emitter–mirror distance d. The fast **a** and intermediate **c** decay components follow the 980-nm excitation enhancement. More specifically, fast decay becomes faster with higher excitation power and intermediate decay becomes slower. The reason for the inverted power dependence of the intermediate decay component is unclear. It is unclear whether the decay rate of the slow component is modulated by the 1550-nm LDOS (solid line) or excitation powers (dashed). **(e)**–**(f)** Fast **e** and slow **f** decay rate of green upconverted emission. The fast component follows the 545-nm LDOS as the rapid NIR decay makes the upconverted dynamics lifetime-limited. The slow component follows the 1550-nm LDOS as the feeding levels decay slower than the emitting level on these timescales. **(g)**–**(h)** Same as **e**–**f**, but for red upconverted emission. Here, the fast component follows the 650-nm LDOS (lifetime-limited) while the slow component follows the 1550-nm LDOS (feeding-limited).

Supplementary references

(S1) Dodson, C.M.; Zia, R. Magnetic dipole and electric quadrupole transitions in the trivalent lanthanide series: calculated emission rates and oscillator strengths *Phys. Rev. B* **2012**, *86*, 125102.

(S2) Karaveli, S.; Zia, R. Spectral tuning by selective enhancement of electric and magnetic dipole emission *Phys. Rev. Lett.* **2011**, *106*, 193004.

(S3) McPeak, K.; Jayanti, S.V.; Kress, S.J.P.; Meyer, S.; Iotti, S.; Rossinelli, A.A.; Norris, D.J. Plasmonic films Can easily be better: rules and recipes *ACS Photonics* **2015**, *2*, 326–333.

(S4) Sokolov, V.I.; Zvyagin, A.V.; Igumnov, S.M.; Molchanova, S.I.; Nazarov, M.M.; Nechaev, A.V.; Savelyev, A.G.; Tyutyunov, A.A.; Khaydukov, E.V.; Panchenko, V.Y. Determination of the refractive index of β-$NaYF_4/Yb^{3+}/Er^{3+}/Tm^{3+}$ nanocrystals using spectroscopic refractometry *Opt. Spectrosk.* **2015**, *118*, 609–613.

(S5) Rabouw, F.T.; Prins, P.T.; Villanueva-Delgado, P.; Castelijns, M.; Geitenbeek, R.G.; Meijerink, A. Quenching pathways in $NaYF_4$:Er^{3+},Yb^{3+} upconversion nanocrystals *ACS Nano* **2018**, *12*, 4812–4823.

(S6) Fischer, S.; Steinkemper, H.; Löper, P.; Hermle, M.; Goldschmidt, J.C. Modeling upconversion of erbium doped microcrystals based on experimentally determined Einstein coefficients *J. Appl. Phys.* **2012**, *111*, 013109.
